# Supplementary material for: Mode of action of teixobactins in cellular membranes
Source: Nat Commun. 2020 Jun 5;11:2848. doi: 10.1038/s41467-020-16600-2 (PMC7275090; doi:10.1038/s41467-020-16600-2)
Supplement: Supplementary file 1 — Supplementary Information [file 41467_2020_16600_MOESM1_ESM.pdf]

Mode of action of teixobactins in cellular membranes

Rhythm Shukla,[1,2] João Medeiros-Silva,[1] Anish Parmar,[3,6,7] Bram J.A. Vermeulen,[1] Sanjit Das,[3,6,7] Alessandra Lucini Paioni,[1] Shehrazade Jekhmane,[1] Joseph Lorent,[2] Alexandre M.J.J. Bonvin,[1] Marc Baldus,[1] Moreno Lelli,[4] Edwin J.A. Veldhuizen,[5] Eefjan Breukink,[2] Ishwar Singh,[3,6,7] Markus Weingarth[1]

[1]NMR Spectroscopy, Bijvoet Centre for Biomolecular Research, Department of Chemistry, Faculty of Science, Utrecht University, Padualaan 8, 3584 CH Utrecht, The Netherlands
[2]Membrane Biochemistry and Biophysics, Bijvoet Centre for Biomolecular Research, Department of Chemistry, Utrecht University, Padualaan 8, 3584 CH Utrecht, The Netherlands
[3]School of Pharmacy, JBL Building, University of Lincoln, Beevor St. Lincoln, UK
[4]Department of Chemistry 'Ugo Schiff', University of Florence, Via della Lastruccia 3, 50019 Sesto Fiorentino (FI), Italy
[5]Section Molecular Host Defence, Division Infectious Diseases & Immunology, Department Biomolecular Health Sciences, Faculty of Veterinary Medicine, Utrecht University, Yalelaan 1, 3584 CL Utrecht, The Netherlands
[6]Present address: Antimicrobial Pharmacodynamics and Therapeutics, Department of Molecular and Clinical Pharmacology, University of Liverpool, Sherrington Building, L69 3GA Liverpool, UK
[7]Present address: Department of Chemistry, The Robert Robinson Laboratories, University of Liverpool, L69 3BX, Liverpool, UK

These authors contributed equally: Rhythm Shukla, João Medeiros-Silva

Correspondence and requests for materials should be addressed to M.W. (email: M.H.weingarth@uu.nl)

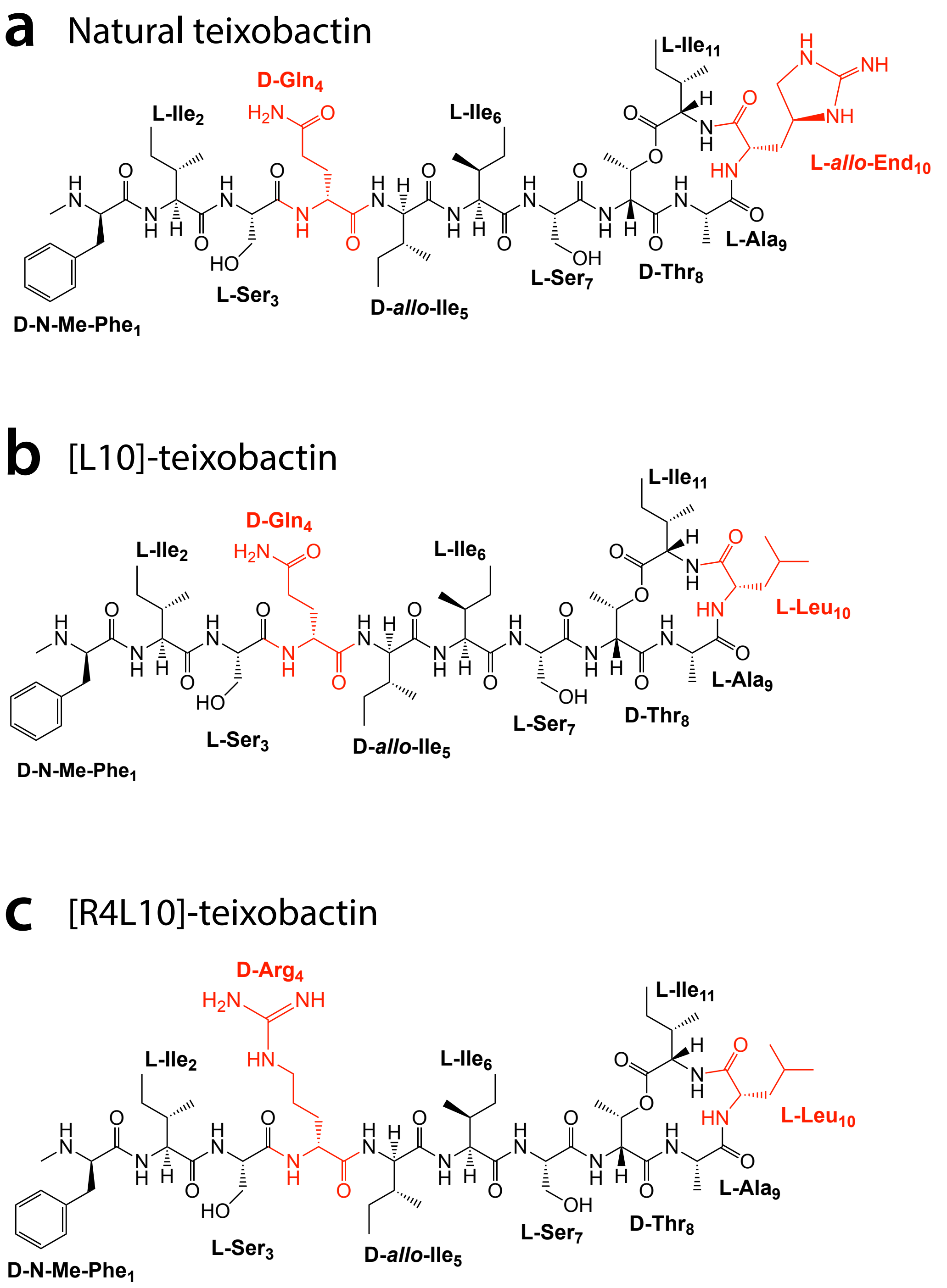

Supplementary Figure 1

Chemical structures of teixobactins. Residues 4 and 10 that were replaced in teixobactin constructs used in this study are highlighted in red. **a)** Natural teixobactin. **b)** [L10]-teixobactin, in which enduracididine at position 10 is replaced by L-Leucine. **c)** [R4L10]-teixobactin, in which enduracididine at position 10 is replaced by L-Leucine, and glutamine at position 4 is replaced by an arginine.

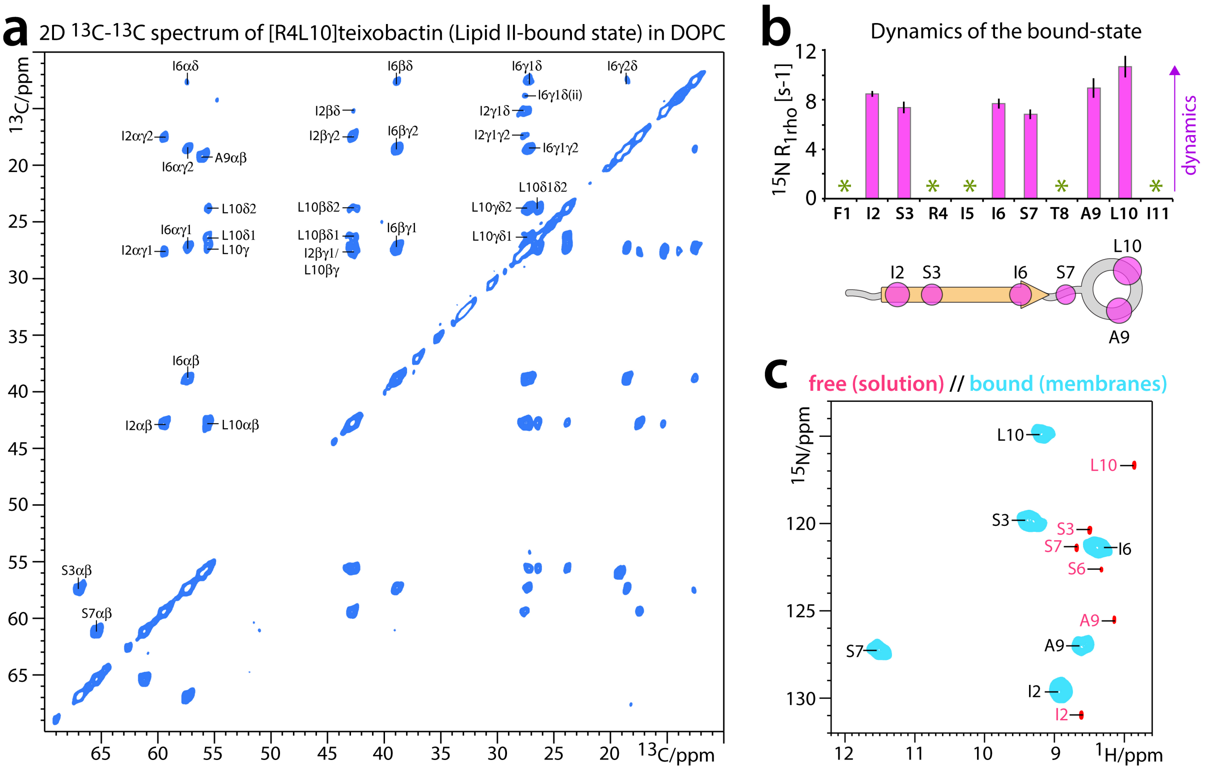

Supplementary Figure 2

[R4L10]-teixobactin forms a well-defined complex with Lipid II in DOPC membranes. All ssNMR measurements were performed at 950 MHz (^{1}H-frequency).
a) Dipolar 2D $^{13}C^{13}C$ spectrum of Lipid II-bound [R4L10]-teixobactin measured with 50 ms $^{13}C^{13}C$ magnetisation transfer time. The high spectral quality and the absence of peak-doubling (expect for a second conformation for the Ile6 sidechain) demonstrate that the complex is well-defined. The spectrum is fully annotated.
b) Site-resolved ^{15}N R_{1rho} ssNMR dynamics of Lysine-Lipid II-bound [R4L10]-teixobactin acquired at 60 kHz magic angle spinning (MAS) in DOPC liposomes. The size of the magenta spheres illustrates the dynamics. Relaxation in the entire molecule is very slow, even slower than in membrane-embedded α-helices of ion channels.[1] The very slow relaxation strongly suggests that the N-terminus is immobilised in a supramolecular interaction. Residues without $^{13}C,^{15}N$-labels are marked by green stars. The error bars show the standard error of the fit. Source data are provided as a Source Data file.
c) Superposition of 2D NH spectra of free [R4L10]-teixobactin in aqueous buffers (in red) and of the Lipid II-bound state in DOPC membranes (cyan). The strong spectral changes demonstrate a well-defined interaction with Lipid II.

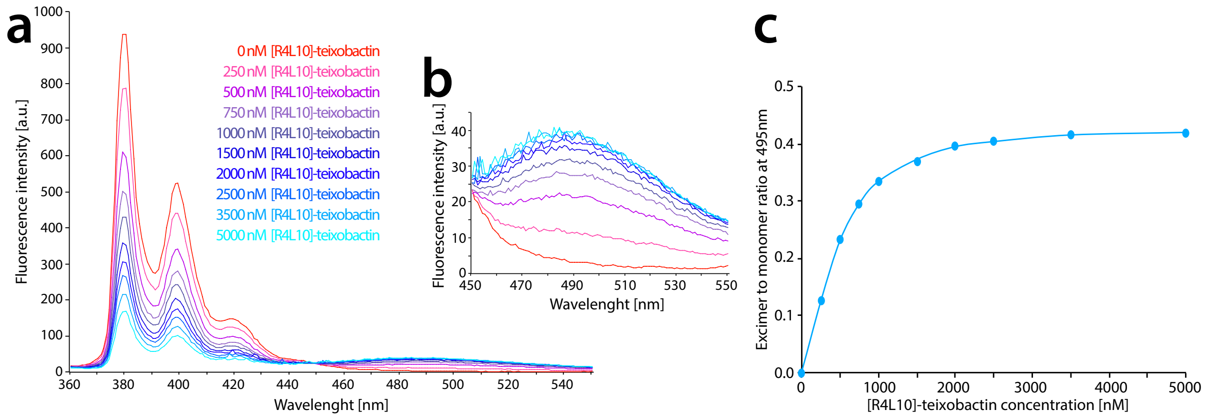

Supplementary Figure 3

Pyrene excimer fluorescence shows that clustered Lipid II molecules bound to teixobactins are within 1 nm of each other in DOPC vesicles.
Monomers of pyrene exhibit characteristic fluorescence emission maxima at 378, 398, and 417 nm. Moreover, pyrene can display a characteristic fluorescence peak at longer wavelengths (490 nm), which occurs only when two pyrene rings reside within 10 Å of each other and form an excited state dimer, which known as an excimer.[2] Source data are provided as a Source Data file.
a) Fluorescence spectra of pyrene-labelled Lipid II symmetrically in large unilamellar vesicles (LUVs) at increasing concentrations of [R4L10]-teixobactin. The spectra show that the fluorescence of the Lipid II monomers goes down while the excimer fluorescence increases. The pyrene fluorescence experiments suggest that the dimerization of teixobactins is almost immediate, and that the oligomerisation into clusters follows from this dimerization.
b) The insert shows the excimer region of plot a).
c) Quantification of the excimer over monomer ratio as a function of the pyrene-labelled Lipid II concentration.

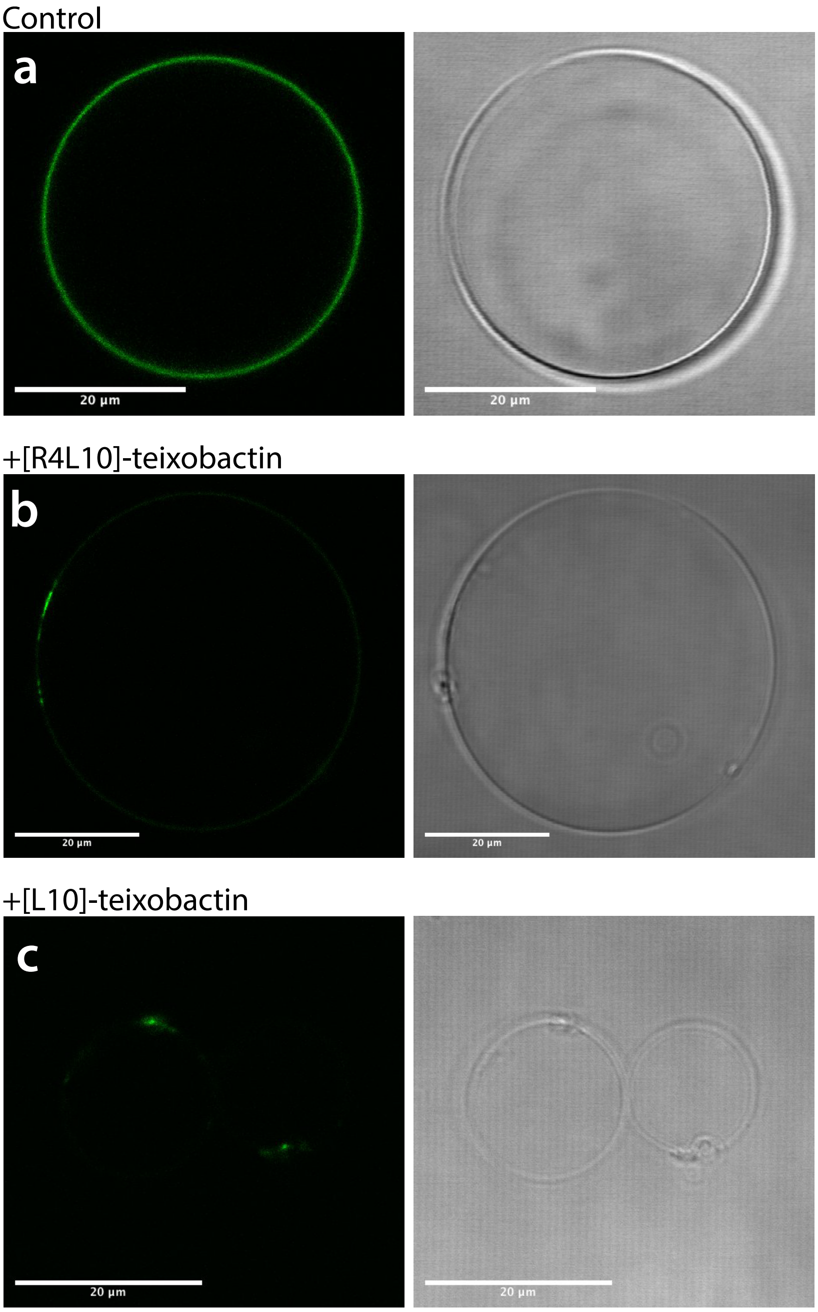

Supplementary Figure 4

Fluorescence microscopy shows that teixobactins sequester Lipid II and delocalise it into clusters on DOPC membranes. Images on the left side show the fluorescence of 7-nitrobenz-2-oxa-1,3-diazol-4-yl (NBD)-labelled Lipid II (488 nm), while the right columns show brightfield images. Images were taken after 4 hours of incubation time, and GUVs were formed from DOPC.

a) The control GUV shows even distribution of NBD-labelled Lipid II on the surface after 4 hours. GUVs incubated with 1µM of **b)** [R4L10]-teixobactin and **c)** [L10]-teixobactin, respectively, for 4 hours. Clustering of Lipid II at certain foci on the surface of the membrane is clearly visible. The brightfield images of the respective GUVs show that the vesicle integrity and morphology is maintained after incubation with teixobactins, although sites of cluster formation show slight membrane deformations.

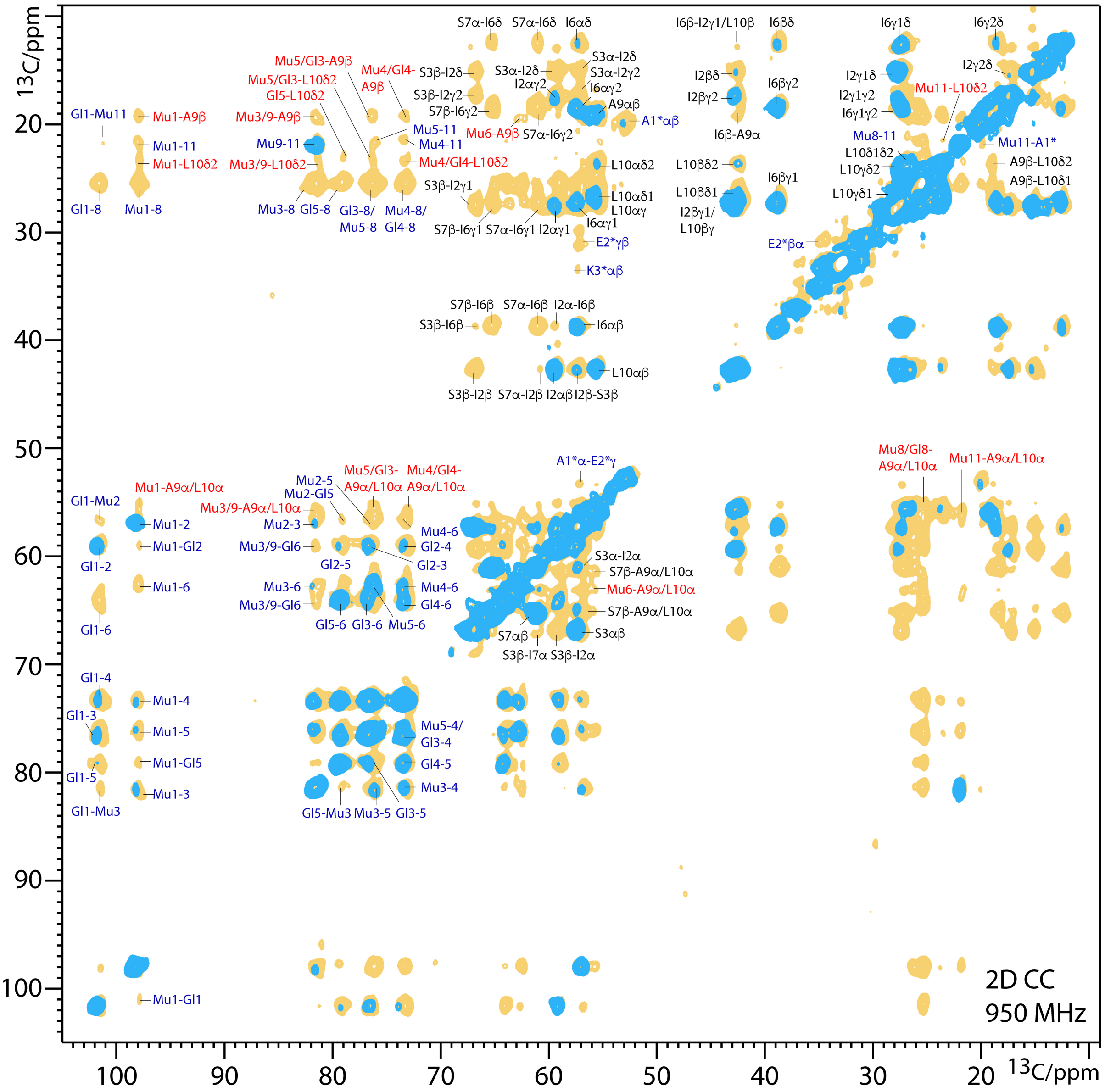

Supplementary Figure 5

[R4L10]-teixobactin – Lipid II interface measured by 2D ^{13}C-^{13}C solid-state NMR in membranes. Spectral overlay of 2D ssNMR spectra of the complex formed between ^{13}C,^{15}N-[R4L10]-teixobactin and ^{13}C,^{15}N-Lipid II. Spectra were acquired at 950 MHz (^{1}H-frequency) and 15.5 kHz magic angle spinning (MAS) using 50 (cyan) and 600 ms (yellow) PARISxy[3] ^{13}C-^{13}C magnetisation transfer time. Contacts within and between [R4L10]-teixobactin molecules are labelled in black, intramolecular Lipid II contacts in blue, and interfacial contacts in red. Residues A1*, E2*, and K3* are from the Lipid II pentapeptide.

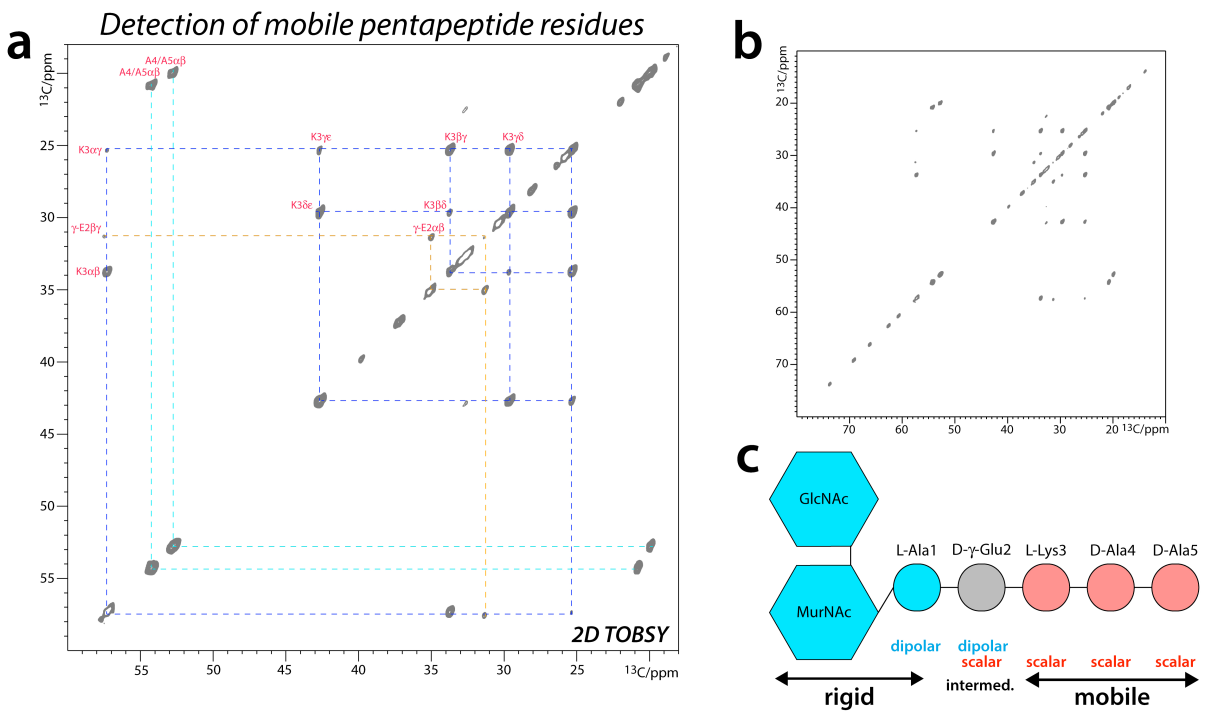

Supplementary Figure 6

The pentapeptide of Lipid II is mobile in the complex. Experiments that rely on so-called scalar couplings can be used to detect residues that show fast nanosecond motion with large amplitudes. I.e., scalar experiments are complementary to experiments that rely on so-called dipolar couplings and which only show rigid residues. **a)** Zoom into a scalar 2D $^{13}C^{13}C$ TOBSY (TOtal through Bond correlation SpectroscopY)[4,5] ssNMR spectrum of the complex formed by $^{13}C,^{15}N$-[R4L10]-teixobactin and $^{13}C,^{15}N$-Lipid II in DOPC liposomes acquired at 950 MHz (^{1}H frequency) magnetic field and 8 kHz MAS and 295 K sample temperature. Lipid II-bound $^{13}C,^{15}N$-[R4L10]-teixobactin and the Lipid II sugars (MurNAc and GlcNAc) are not visible in the spectrum, demonstrating that they are rigid in the complex. Conversely, the last three residues (Lys3-Ala4-Ala5) of the Lipid II pentapeptide give strong scalar signals, while γ-Glu2 shows gives clear but weaker scalar signals. Ala1, which is directly bound to the rigid MurNAc, is only visible in dipolar-based spectra (see Fig. 2d of the manuscript). **b**) A larger spectral region of the 2D $^{13}C^{13}C$ TOBSY spectrum. No sugar signals could be detected, demonstrating that both MurNAc and GlcNAc sugars are rigid in the complex. **c)** The mobility profile of the pentapeptide of [R4L10]-teixobactin-bound Lipid II.

In our TOBSY sequence, the basic recoupling pulse cycle ($90_x - 360_{-x} - 270_x$) needs to be repeated three times during one rotor period in order to suppress anisotropic NMR interactions. This led to a ^{13}C power requirement of six times the MAS frequency. Therefore, we used slow MAS (8 kHz) for this experiment.

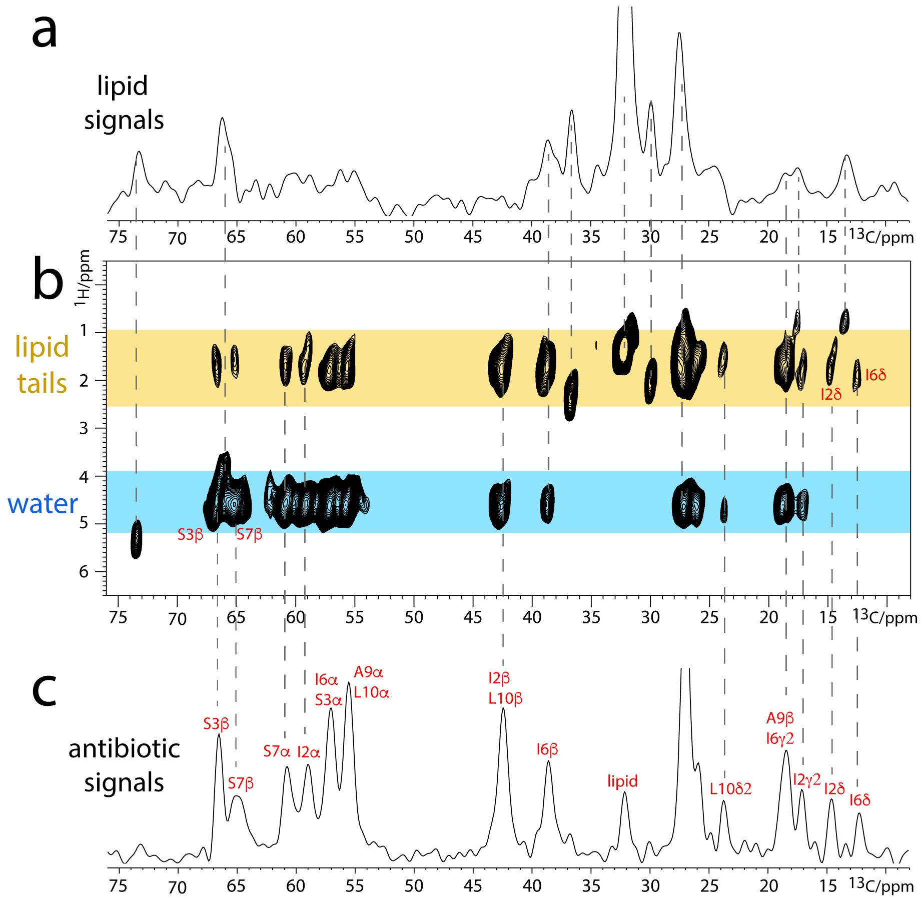

Supplementary Figure 7

[R4L10]-teixobactin localises at the water-lipid interface while hydrophobic sidechains act as membrane anchors. a) and b) show motion-edited ^{1}H(^{1}H)^{13}C ssNMR experiments.[6,7] In these experiments, application of a T_2-relaxation filter destroys magnetization of the rigid complex while magnetization on mobile water and lipid molecules is maintained and subsequently transferred to the rigid Lipid II-bound [R4L10]-teixobactin via ^{1}H-^{1}H mixing. The magnetization is eventually transferred to the ^{13}C nuclei of the rigid antibiotic via a short (200 us) dipolar cross polarisation step. A 2D implementation of this experiment enables to measure to which extent residues partition into water and lipid phases. The 2D experiment unambiguously shows that the sidechains of Ile2 and Ile6 partition into the lipid phase. All spectra were measured at 700 MHz (^{1}H-frequency), 16.5 kHz MAS, and at 300 K sample temperature.

a) 1D mobility-edited ^{1}H(^{1}H)^{13}C ssNMR spectrum of [R4L10]-teixobactin bound to Lipid II measured at 700 MHz using a T_2 relaxation delay of 2.5 ms to de-phase signals of the complex. ^{1}H-^{1}H mixing was set to zero, i.e., no magnetization was transferred to the rigid complex, and all visible signals relate to residual lipid signals, demonstrating the effectiveness of the T_2 mobility filter. The spectrum was acquired with 10240 scans. **b)** 2D mobility-edited ^{1}H(^{1}H)^{13}C ssNMR spectrum of [R4L10]-teixobactin bound to Lipid II measured at 700 MHz using a T_2-relaxation filter of 2.5 ms. Subsequently, 5 ms ^{1}H-^{1}H mixing was used to transfer magnetization from mobile water and lipid tails to the complex. The 2D spectrum was measured with 8192 scans. **c)** ^{13}C cross-polarization spectrum (200 us contact time) of [R4L10]-teixobactin bound to Lipid II measured at 700 MHz magnetic field.

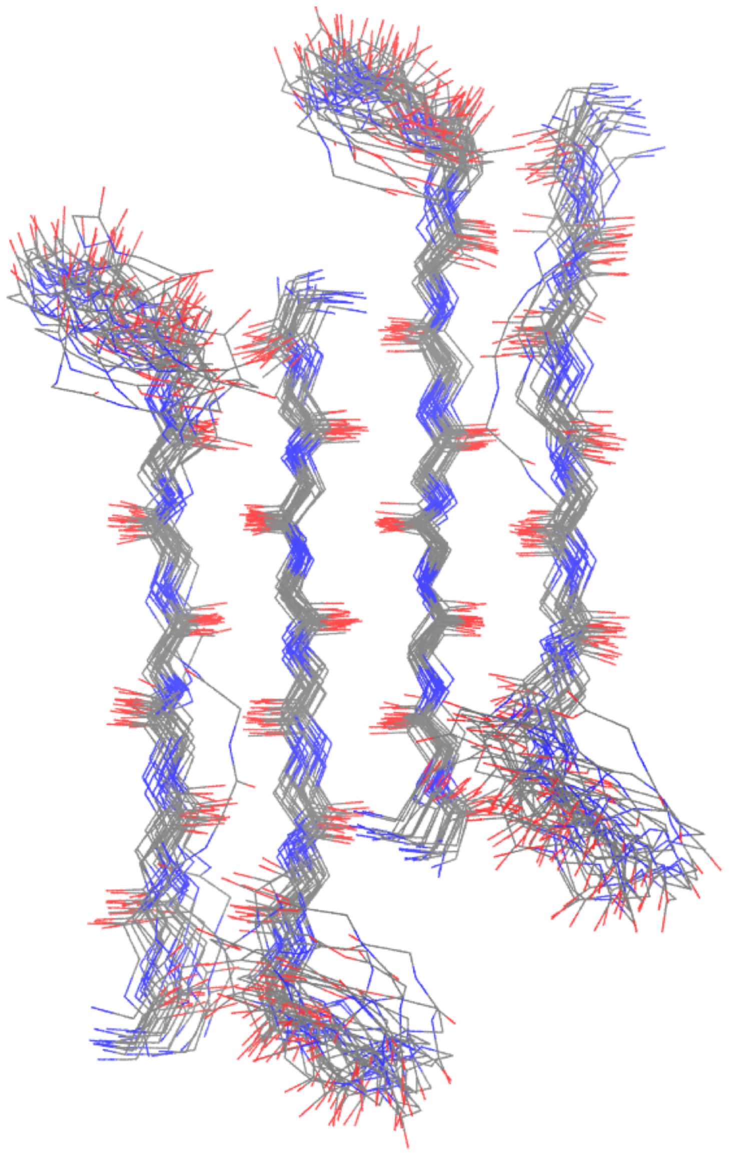

Supplementary Figure 8

Structures of the drug in the complex superimpose well. Superposition of 26 structures of four [R4L10]-teixobactin peptides bound to four Lipid II molecules (not shown). The backbone RMSD is 1.77 +/- 0.49 Å (see also methods section).

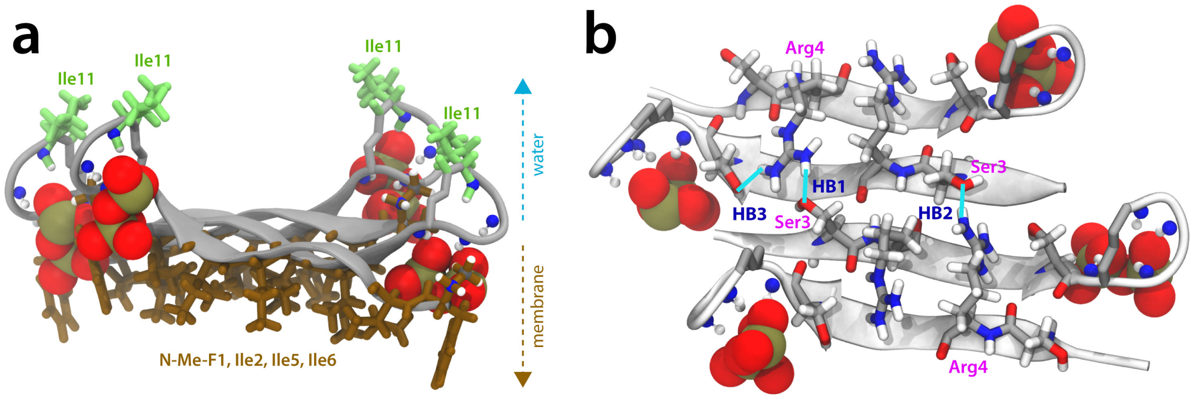

Supplementary Figure 9

Interactions on the water-exposed part of the β-sheet formed by Lipid II-bound [R4L10]-teixobactin.

a) The long hydrophobic sidechains of D-N-Me-1, Ile2, D-allo-Ile5, and Ile6 act as essential membrane anchors, whose replacement is not tolerated.[8-10] Ile11 is water-exposed in the complex, and its replacement by alanine is tolerated.[10]

b) The long sidechain of the arginine at position 4 enables hydrogen bonds over three monomers.

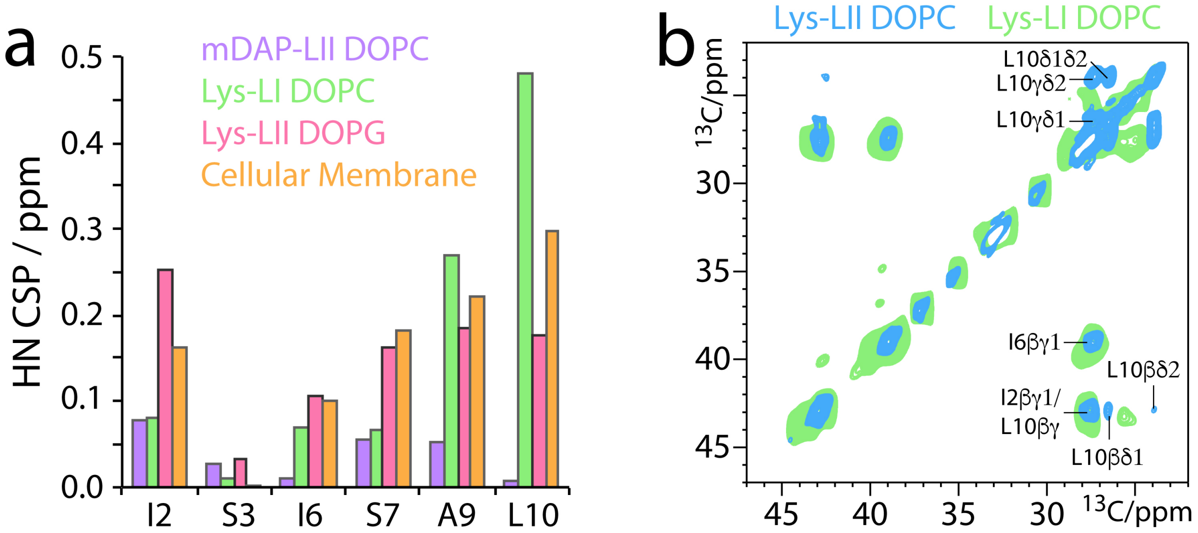

Supplementary Figure 10

Impact of receptor structure and membrane composition on receptor bound-[R4L10]-teixobactin. a) Combined (^{1}H+^{15}N) chemical shift perturbations (HN CSPs) of the spectra shown in Fig. 4a of the manuscript in reference to Lys-Lipid II in DOPC liposomes. HN CSPs (in ppm) were calculated according to

$$CSP_{HN} = \sqrt{(\Delta H)^2 + \frac{(\Delta N)^2}{6.5}}$$

Source data are provided as a Source Data file.

b) Superposition of dipolar 2D solid-state NMR $^{13}C^{13}C$ spectra of [R4L10]-teixobactin bound to Lys-Lipid II (blue) and Lys-Lipid I (green), showing a conformational change of the Leu10 sidechain. Both spectra were acquired in DOPC membranes. The signal shifts of the Leu10 sidechain show that GlcNAc sugar of Lipid II modulates, presumably allosterically, the binding interface.

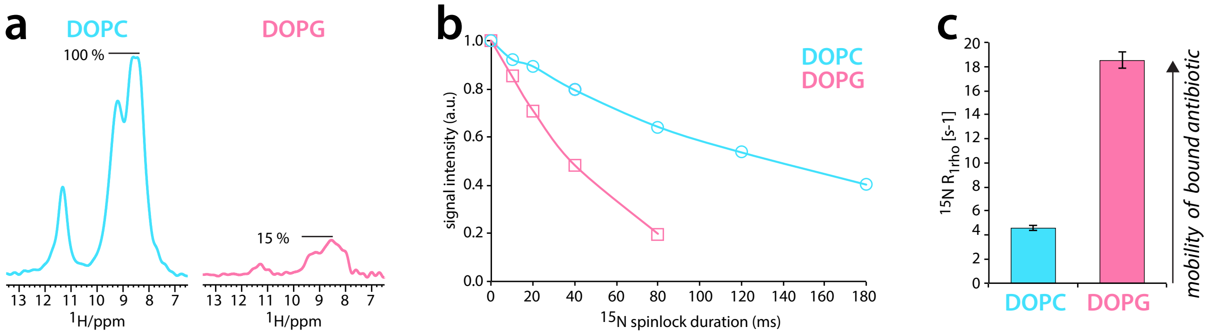

Supplementary Figure 11

Weaker binding affinity to Lipid II in anionic conditions is a common property of the binding mode of teixobactins.
To validate and generalise our findings, we repeated ssNMR studies with a second analogue, [L10]-teixobactin, which is close to natural teixobactin. Both [L10]-teixobactin and [R4L10]-teixobactin show similar behaviour in anionic membranes, i.e., sharp drops in ssNMR sensitivity and marked increase in mobility in the complex. We note that only the difference between natural teixobactin and [Leu10]-teixobactin is the substitution of enduracididine10 by leucine10. We also note that the substitution by enduracididine10 by cationic or hydrophobic residues is tolerated and does not or only modestly change the activity of teixobactins.[8,11-13]

a) ^{1}H-detected 1D NH spectra of Lipid II-bound [Leu10]-teixobactin measured in DOPC (cyan) and DOPG (red) liposomes. The signal-to-noise ratio is more than six times lower in anionic membranes. Both spectra were acquired with fully packed 1.3 mm rotors and 10240 scans each.
b)^{15}N $T_{1\mathrm{rho}}$ signal decay curves of the bulk signal of Lipid II-bound [Leu10]-teixobactin in DOPC (cyan) and (DOPG (red) membranes. Relaxation is much faster in anionic membranes, demonstrating enhanced mobility. Source data are provided as a Source Data file.
c) Corresponding ^{15}N $R_{1\mathrm{rho}}$ rates of Lipid II-bound [Leu10]-teixobactin in DOPC (cyan) and DOPG (red) membranes. The error bars show the standard error of the fit. Source data are provided as a Source Data file.

The spectra were acquired at 700 MHz with 60 kHz magic angle spinning (MAS).

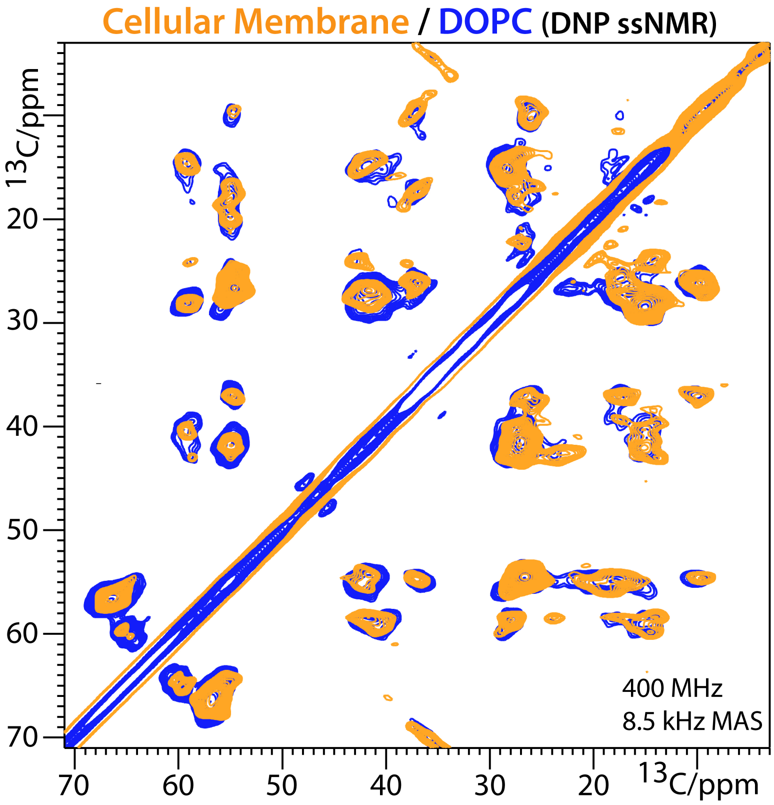

Supplementary Figure 12

The structure of the bound antibiotic is similar in synthetic DOPC liposomes and in cellular *M. flavus* membranes. DNP-ssNMR 2D $^{13}C^{13}C$ spectra of Lipid II-bound [R4L10]-teixobactin in DOPC (blue) and *M. flavus* membranes (orange). The good match between the two spectra shows that the structure of the complex is conserved in natural membranes. Moreover, the unusually narrow signals in the DNP-ssNMR spectrum, acquired at cryogenic temperatures (100 K) at which conformational dynamics would manifest in marked signal-broadening, show that the complex is well-defined under cellular conditions. Both DNP spectra were acquired at 400 MHz/263 GHz using 8.5 kHz MAS with 20 ms $^{13}C^{13}C$ magnetization transfer time. The DNP enhancement factor was approximately 50.

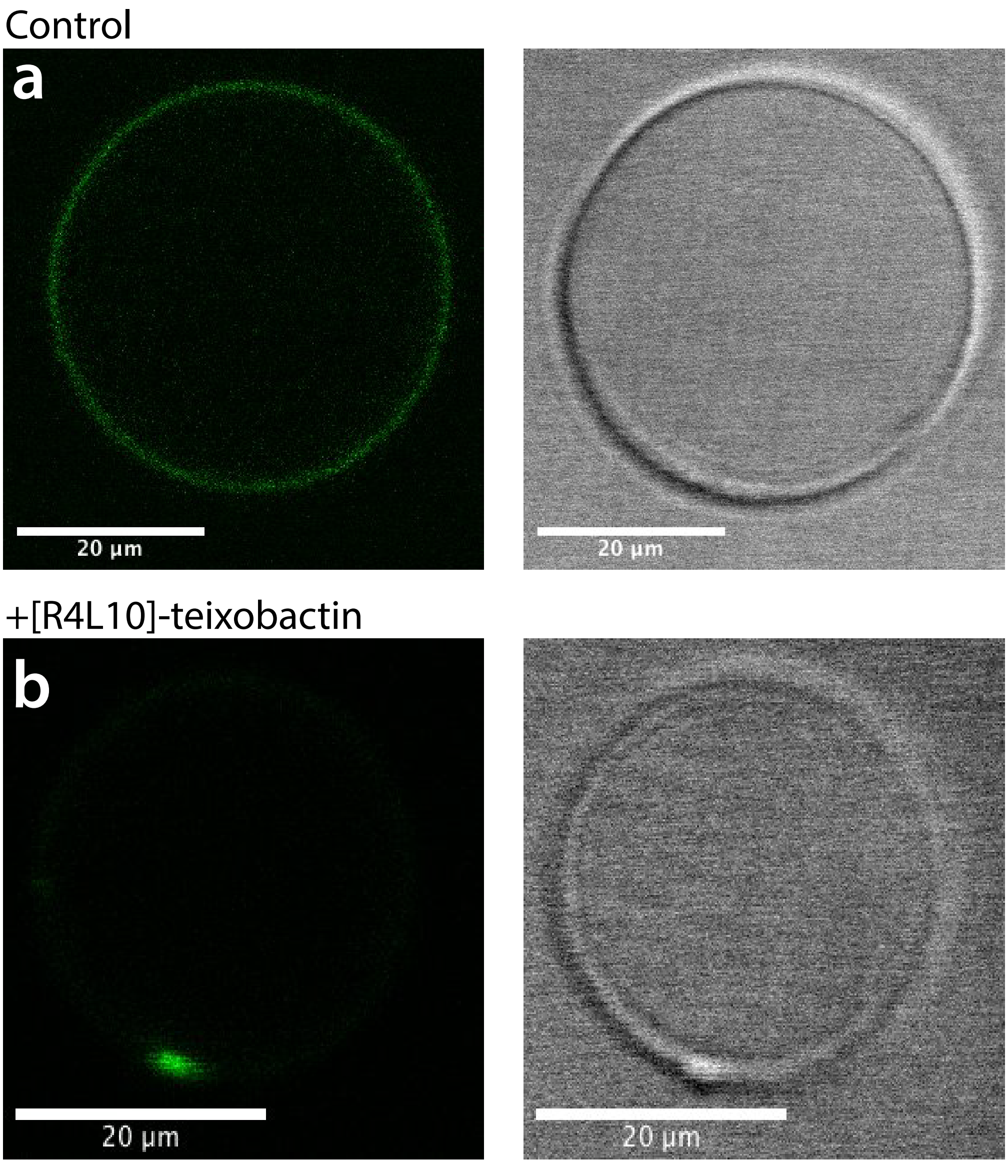

Supplementary Figure 13

Fluorescence microscopy shows that teixobactins effectively capture Lipid II in clusters in anionic membranes. Images on the left side show the fluorescence of NBD-labelled Lipid II (488 nm), while the right columns show brightfield images. All images were taken after 4 hours of incubation time. Anionic GUVs were formed from a 1:3 DOPG:DOPC (mol/mol) mixture doped with 0.1 mol% NBD-labelled Lipid-II.

a) The control GUVs show even distribution of NBD-labelled Lipid II on the surface.

b) Anionic GUVs incubated with 1µM of [R4L10]-teixobactin. Clustering of Lipid II at certain foci on the surface of the membrane is clearly visible. Clustering is not compromised in anionic membrane, despite the markedly reduced binding affinity of teixobactins to Lipid II in these conditions (see Figure 4d,e of the main text).

Supplementary Table T1: *$^{13}C,^{15}N$-labelled-(I2,S3,I6,S7,A9,L10)-[R4L10]-teixobactin in the Lipid-II bound state* / Measurements in DOPC liposomes.*

Res	**#**	**NH**	**HN**	**CO**	**CA**	**CB**	**CG1**	**CG2**	**CD**	**CD2**
**Ile**	**2**	*129.52*	*8.99*	*172.75*	*59.16*	*42.43*	*27.31*	*16.85*	*14.97*	
**Ser**	**3**	*119.74*	*9.35*	*172.47*	*57.14*	*66.69*				
**Ile**	**6**	*121.25*	*8.45*	*176.18*	*57.26*	*38.51*	*26.82*	*18.11*	*12.26*	
**Ser**	**7**	*127.09*	*11.57*	*176.96*	*61.01*	*65.07*				
**Ala**	**9**	*126.81*	*8.6*	*178.02*	*55.80*	*18.57*				
**Leu**	**10**	*114.76*	*9.25*	*176.05*	*55.38*	*42.52*	*27.20*		*26.44*	*23.51*

*assignments in ppm

Supplementary Table T2: *$^{13}C,^{15}N$-labelled-Lipid II, bound-state** / Measurements in DOPC liposomes*

**Sugars**	**#**	**C1**	**C2**	**C3**	**C4**	**C5**	**C6**	**C7**	**C8**	**C9**	**C10**	**C11**
**MurNAc**		*97.91*	*56.52*	*81.47*	*73.24*	*76.11*	*62.43*	*176.51*	*25.47*	*81.27*	*178.4*	*21.59*
**GlcNAc**		*101.4*	*58.83*	*76.29*	*73.33*	*76.06*	*64.1*	*176.51*	*25.47*			
Pentapep		**CO**	**CA**	**CB**	**CG**	**CD**	**CE**					
**L-Ala**	**1**	*176.86*	*52.85*	*19.82*								
**D-γ-Glu**	**2**		*34.97*	*31.32*	*57.52*	*180.31*						
**L-Lys**	**3**		*57.3*	*33.36*	*25.35*	*29.67*	42.69					
**D-Ala**	**4****		*54.31*	*20.82*								
**D-Ala**	**5****		*52.81*	*19.97*								

*assignments in ppm.
**Assignments for residues Ala4 and Ala 5 of the pentapeptide could be in inverse order.

Supplementary Table T3: Number and type of restraints used for the structure calculations. Intramolecular restraints are listed per monomers, while intermolecular/interfacial restraints are listed per pair of interacting molecules.

Number of Restraints		
Intramolecular	*Unambiguous*	*Ambiguous*
[R4L10]-teixobactin distance restrains	5	2
[R4L10]-teixobactin dihedral restrains*	12	0
Lipid II distance restraints	11	10
Intermolecular ***[R4L10]-teixob. - [R4L10]-teixob.***	*Unambiguous*	*Ambiguous*
Distance restraints	9	8
Hydrogen bonds**	6	0
Intermolecular ***[R4L10]-teixobactin - Lipid II***	*Unambiguous*	*Ambiguous*
Distance restraints with sugars	5	13
Distance restraints with PPI***	0	4

*,**,*** See Methods (NMR Structure calculations) for details

Supplementary Methods

NMR restraints and Structure validation

Intramolecular [R4L10]-teixobactin distance restraints: We defined intramolecular restraints between residues Ser7 to residues Ala9 and Leu10 based on 2D $^{13}C^{13}C$ PARIS experiments with different magnetization transfer times using upper and lower limits of 7.0 and 2.0 Å, respectively (8.0 and 3.0 Å for few weak signals).

Dihedral angle restraints (intramolecular): Dihedral angle restraints were applied for Ile2-Ile7 of [R4L10]-teixobactin. Since the N-terminus of [R4L10]-teixobactin, [L10]-teixobactin, and natural teixobactin[14,15] form β-sheet structures, we extracted average dihedral angle from the X-ray structure of a teixobactin in complex with a sulphate ion, where the N-terminus has β-strand conformation.[16] Restraints were implemented with boundaries of +/- 20°. We validated this procedure by deriving dihedral angles from our ssNMR assignments using the TALOS+[17] software. Indeed, ssNMR-based dihedrals could be obtained for residues Ile2 and Ile6 (with 'Good' accuracy, i.e., the best accuracy), which matched well to the N-terminus of the X-ray structure.

Intramolecular Lipid II distance restraints: Contacts within and between the MurNAc and GlcNAc sugar were defined with upper and lower limits of 6.0 and 1.5 Å, respectively, based on a series of 2D $^{13}C^{13}C$ PARIS.

Intermolecular distance restraints between [R4L10]-teixobactin molecules
All restraints based on a series of 2D $^{13}C^{13}C$ PARIS experiments using upper and lower limits of 7.0 and 2.0 Å, respectively (8.0 and 3.0 Å for few weak signals). These restraints were fulfilled in all 26 structures.

Hydrogen bond restraints (intermolecular) between [R4L10]-teixobactin molecules: Hydrogen bonding restraints in-line with experimentally determined anti-parallel *[R4L10]-teixobactin* β-sheets were applied. Antiparallel β-sheets were established by ssNMR (Fig. 1d,e,f of the main manuscript) and validated by fluorescence spectroscopy. While three different register shifts are possible to form antiparallel [R4L10]-teixobactin β-sheets (i.e., Ser3NH could interact with Ser7CO, Ile5CO, or Ile3CO), only the variant with Ser7CO agrees with the intermolecular ssNMR distance restraints. Hydrogen bonding distance restraints were defined accordingly with upper and lower limits of 2.3 and 1.5 Å, respectively.

[R4L10]-teixobactin ***A*** *with [R4L10]-teixobactin* ***B***

Ser3NH	-	Ser7CO
Ser3CO	-	Ser7NH
Ile5NH	-	Ile5CO
Ile5CO	-	Ile5NH
Ser7NH	-	Ser3CO
Ser7CO	-	Ser3NH

[R4L10]-teixobactin ***B*** *with [R4L10]-teixobactin* ***C***

Ile2NH	-	Ile6CO
Ile2CO	-	Ile6NH
Arg4NH	-	Arg4CO

Arg4CO	-	Arg4NH
Ile6NH	-	Ile2CO
Ile6CO	-	Ile2NH

*[R4L10]-teixobactin **C** with [R4L10]-teixobactin **D***
Similar as in ***A*** with ***B***

Intermolecular distance restraints between [R4L10]-teixobactin and Lipid II: *Restraints involving the pyrophosphate group:* Ambiguous distance restraints were applied between the backbone amino protons of the four ring residues (Thr8-Ile11) with either phosphate of the pyrophosphate group using upper and lower limits of 2.4 and 1.7 Å, respectively. Restraints based on a 2D $^{1}H^{31}P$ experiment (Fig. 2b of the manuscript). *Restraints involving the MurNAc and GlcNAc sugars:* Restraints were applied using boundaries of using upper and lower limits of 8.0 and 1.5 Å, respectively (9.0 and 2.0 Å for few weak signals). Restraints were based on a series of 2D $^{13}C^{13}C$ PARIS experiments

Topological restraints: Eventually, a filtering strategy was applied. Structures were only accepted if all Lipid II tails pointed into the direction of the membrane-anchoring residues Ile2, Ile5, and Ile6) (see Fig. 2f of the main text and Supplementary Figure 7). Sorting of the Lipid II tails was steered by imposing distance restraints between the sidechain of Ile6 and the Lipid II isoprenyl-tail.

Analysis of calculated structures
Structural and violation statistics of the final 26 structures are given below:

Structure ensemble precision:

- Average backbone RMSD (from the average structure) of the 26 [R4L10]-teixobactin molecules in the complex: 1.77 +/- 0.49 Å.

Violation analysis:

- *Intramolecular Lipid II distance restraints:* No single violation in all 4 x 26 = 104 Lipid II monomers.

- *Intramolecular teixobactin restraints:*
    - Distance restraints S7Cα - L10Cd1 and S7Cβ - L10Cd1 violated in <20 of 104 (<20 %) of *[R4L10]-teixobactin* monomers.
    - 11 of 12 dihedral restraints fulfilled in 95 % of monomer structures. The Psi restraint of Ser7 was modestly violated on average by +13° degrees in 57 of the 104 monomer structures.

- *Intermolecular [R4L10]-teixobactin- [R4L10]-teixobactin restraints*

- Distance rest. >99 % fulfilled. One rest. violated in 1 of 26 structures.
- Hydrogen bond restraints 100 % fulfilled in all 26 structures

- Intermolecular *[R4L10]-teixobactin* – Lipid II restraints

    - Distance restraints between Thr8/Ala9 and PPi fulfilled for all pairs. Restraints between Leu10NH and PPi fulfilled for 100 of 104 pair. Distance restraint of Ile11NH with PPi was violated in 58 % of pairs by on average 1.2 Å. This was the only pronounced violation in the complex structures.
    - 15 out of 17 distance restraints between [R4L10]-teixobactin – Lipid II were fulfilled for >95% of the pairs. Restraint between L10Cδ2 and MurNAcC11 was violated in 23 of 104 pairs (22 %); restraint between A9/L10Cα was violated for 25 of 104 pairs (24 %).

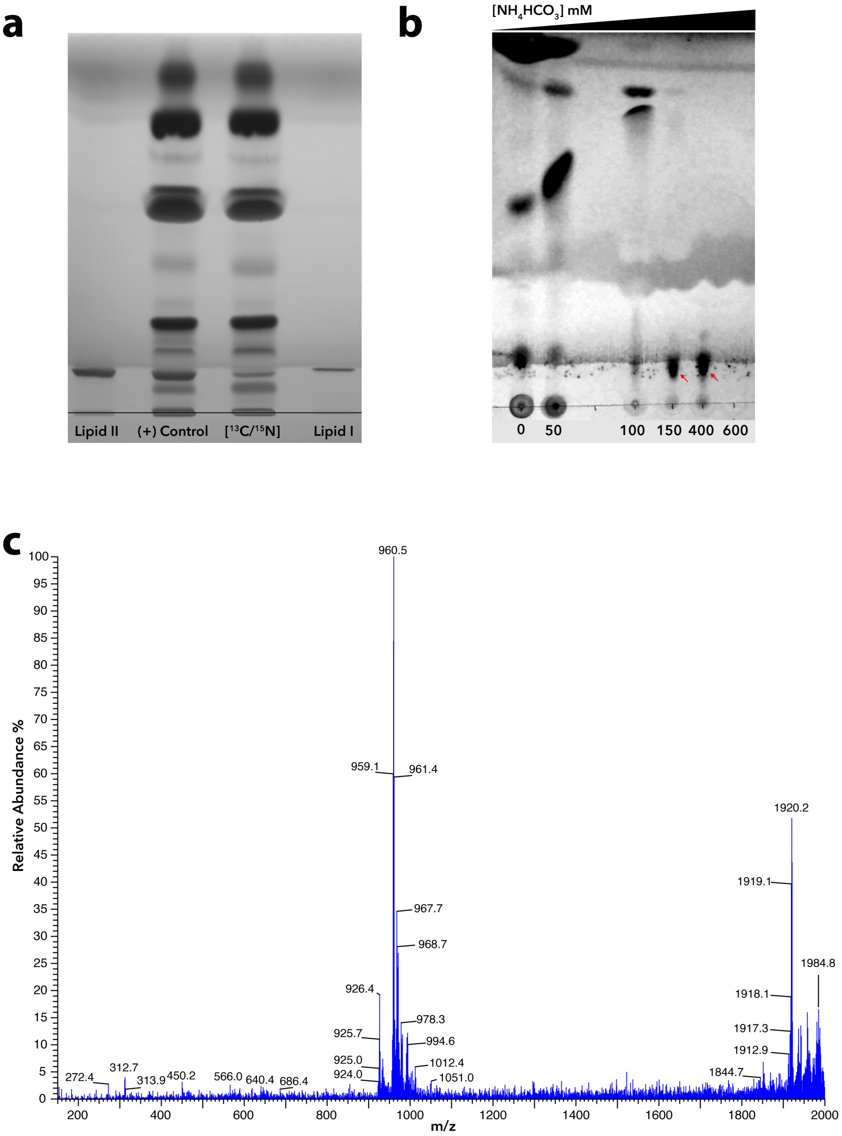

Supplementary Figure 14| a) Thin Layer Chromatography (TLC) of the unlabelled (centre-left) and labelled (centre-right) crude Lipid II extract. The result shows the production of head-group labelled Lipid II with the precursors obtained from labelled *S. Simulans*. **b)** Lipid II, annotated with an arrow, was purified via anion exchange chromatography. The presence of Lipid I species is negligible. **c)** ESI ionization trace (positive mode) of the 400 mM fraction from B). The calculated molecular weight of [^{13}C/^{15}N] head-group-labelled Lipid II is 1920 Da, which matches the observed mass values [$M+H^+$] = 1920.2 Da, [$M/2+H^+$] = 960.5 Da). The major isoprenyol chain species corresponds to 11 isoprenol units, although minor populations of 10 and 12 isoprenol units are also present (MW of 1854 and 1986 Da, respectively), which is consistent with previous studies.[18]

Syntheses of teixobactins

Teixobactin labelled analogues were synthesised using adapted protocols from our previously reported synthesis (Supplementary Figures 15, 16, and 17).[19] For simplicity, in this paper D-Arg$_4$-Leu$_{10}$-teixobactin is represented as [R4L10]-teixobactin and Leu$_{10}$-teixobactin as [L10]-teixobactin.

Synthesis of ^{13}C,^{15}N-I2,S3,I6,S7,A9,L10-labelled [R4,L10]-teixobactin:

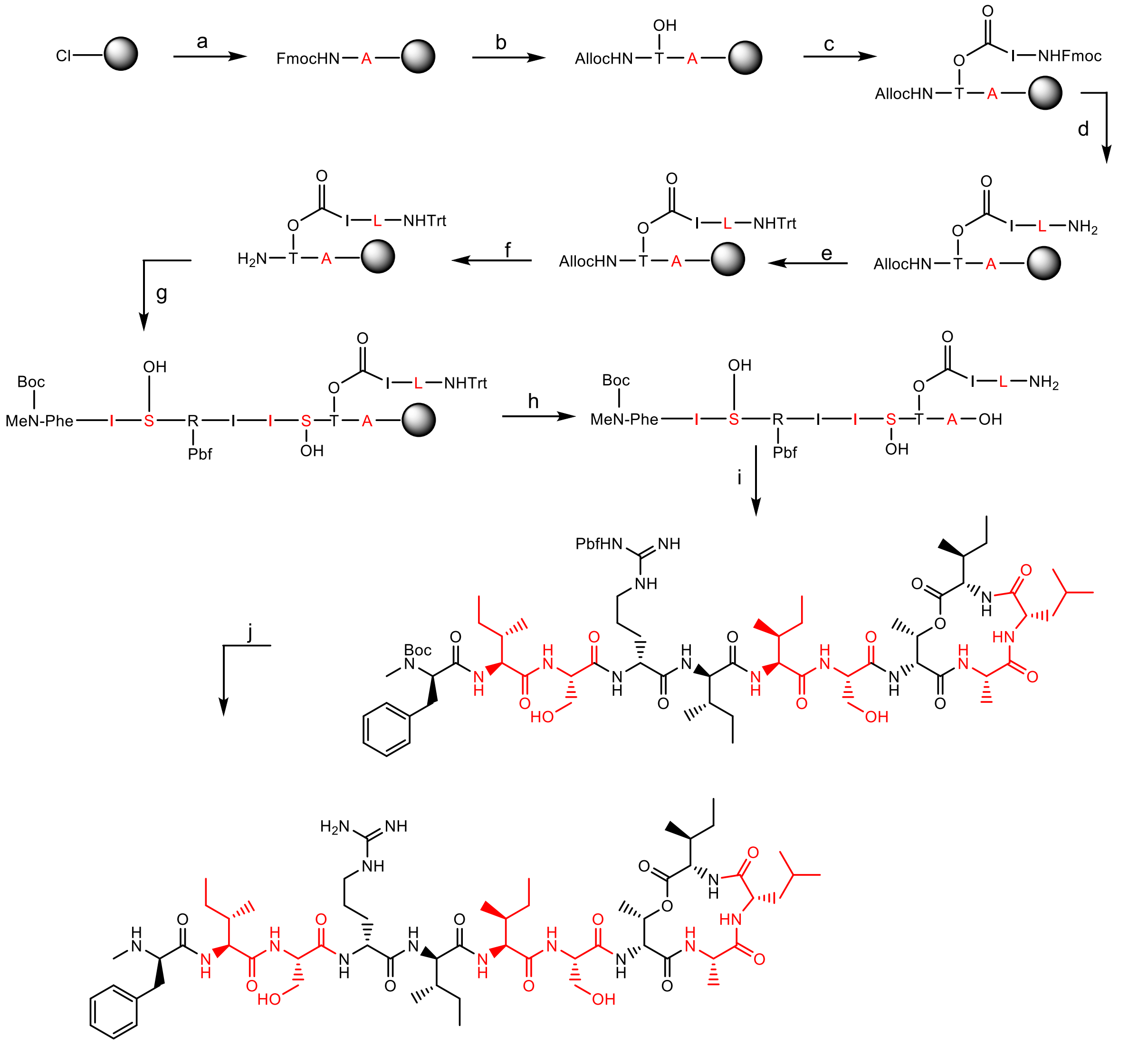

Supplementary Figure 15| Synthesis of labelled D-Arg$_4$-Leu$_{10}$-teixobactin (2). Amino acids in red denote ^{13}C and ^{15}N labeled atoms.

(step a) Commercially available 2-Chlorotrityl chloride resin (manufacturer's loading = 1.6 mmol/g, 150 mg resin) was swelled in DCM in a reactor. To this resin was added 4 eq. Fmoc-Ala-OH/8 eq. DIPEA in DCM and the reactor was shaken for 3h. The loading determined by UV absorption of the piperidine-dibenzofulvene adduct was calculated to be 0.56 mmol/g, (150mg resin, 0.084 mmol). Any unreacted resin was capped with MeOH:DIPEA:DCM = 1:2:7 by shaking for 1h. (step b) The Fmoc protecting group was deprotected using 20% piperdine in DMF by shaking for 3 min, followed by draining and shaking again with 20% piperidine in DMF for 10 min. AllocHN-D-Thr-OH was then coupled to the resin by adding 3 eq. of the AA, 3 eq. HATU and 6 eq. DIPEA in DMF and shaking for 1.5h at room temperature. (step c) Esterification was performed using 10 eq. of Fmoc-Ile-OH, 10 eq. DIC and 5 mol% DMAP in DCM and shaking the reaction for 2h. This was followed by capping the unreacted alcohol using 10% Ac_2O/DIPEA in DMF shaking for 30 min and Fmoc was removed using protocol described earlier in step (b). (step d) Fmoc-Leu-OH was coupled using 4 eq. of AA, 4 eq. HATU and 8 eq. DIPEA in DMF and shaking for 1h followed by Fmoc deprotection using 20% piperidine in DMF as described earlier.

(step e) The N terminus of Leu was protected using 10 eq. Trt-Cl and 15% Et_3N in DCM and shaking for 1h. The protection was verified by the Ninhydrin colour test. (step f) The Alloc protecting group of D-Thr was removed using 0.2 eq. $[Pd(PPh^3)]^0$ and 24 eq. $PhSiH_3$ in dry DCM under argon for 20 min. This procedure was repeated again increasing the time to 45 min and the resin was washed thoroughly with DCM and DMF to remove any excess Pd stuck to the resin. (step g) All amino acids were coupled using 4 eq. Amino Acid, 4 eq. HATU and 8 eq. DIPEA for 1hr. (step h) The peptide was cleaved from the resin without cleaving off the protecting groups of the amino acid sidechains using TFA:TIS:DCM = 2:5:93 and shaking for 1h. (step i) The solvent was evaporated and the peptide was dissolved in DMF to which 1 eq. HATU and 10 eq. DIPEA were added and the reaction was stirred for 30 min to perform the cyclisation. (step j) The side-chain protecting groups were then cleaved off using TFA:TIS:H2O = 95:2.5:2.5 by stirring for 1h. The peptide was precipitated using cold Et_2O (-20°C) and centrifuging at 7000 rpm to obtain a white solid. This solid was further purified using RP-HPLC using the protocols described previously (Supplementary Figure 16c,d).[19] The teixobactin analogues (**1**-**2**) were identified by MS in positive mode (Supplementary Table T4), (Supplementary Figure 16e,f)

Supplementary Table T4*:* Compound number, name, chemical formula, exact mass and mass found for compounds **1-2.** The overall yields were compound 1 (15%) and Compound 2 (18%) respectively.

**Compound**	**Name**	**Chemical formula**	**Exact mass**	**Mass found [M +H⁺]**
**1**	Leu_{10}-teixobactin- ^{13}C & ^{15}N (Ile_2, Ser_3, Ile_6, Ser_7, Ala_9, Leu_{10})	$C_{58}H_{96}N_{12}O_{15}$	1233.71	1234.81
**2**	$_D$-Arg_4-Leu_{10}-teixobactin- ^{13}C & ^{15}N (Ile_2, Ser_3, Ile_6, Ser_7, Ala_9, Leu_{10})	$C_{59}H_{100}N_{14}O_{14}$	1261.75	1262.77

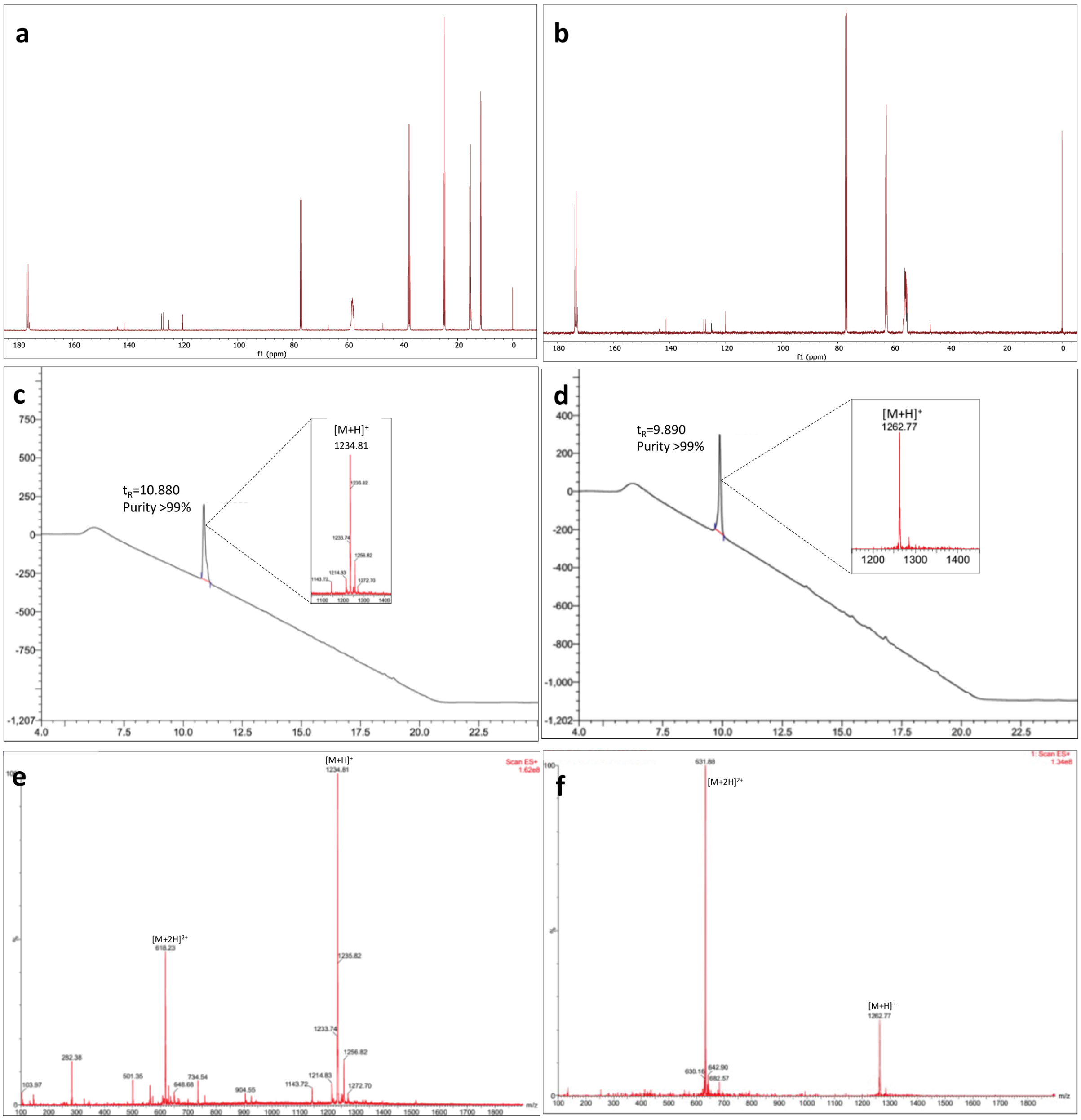

Supplementary Figure 16| Characterisation of labelled amino acids (compounds S1 & S2) and labelled peptides (analogues 1 & 2). a) ^{13}C NMR Spectra of compound S1. **b**) ^{13}C NMR Spectra of compound S2. **c**) HPLC chromatogram of **L10-teixobactin (1)**, in inset, trace of MS spectra of teixobactin analogue **1** showing a peak at m/z 1234.81 corresponding to $[M+H]^+$. **d**) HPLC chromatogram of **[R4L10]-teixobactin (2)**, in inset, trace of MS spectra of teixobactin analogue **2** showing a peak at m/z 1262.77 corresponding to $[M+H]^+$. **e**) MS spectra of teixobactin analogue **1** showing a peak at m/z 1234.81 and 618.23 corresponding to $[M+H]^+$ and $[M+2H]^{2+}$ respectively. **f**) MS spectra of teixobactin analogue **2** showing a peak at m/z 1262.77 and 631.88 corresponding to $[M+H]^+$ and $[M+2H]^{2+}$, respectively.

Syntheses of ^{13}C,^{15}N-labelled Fmoc-amino acids:

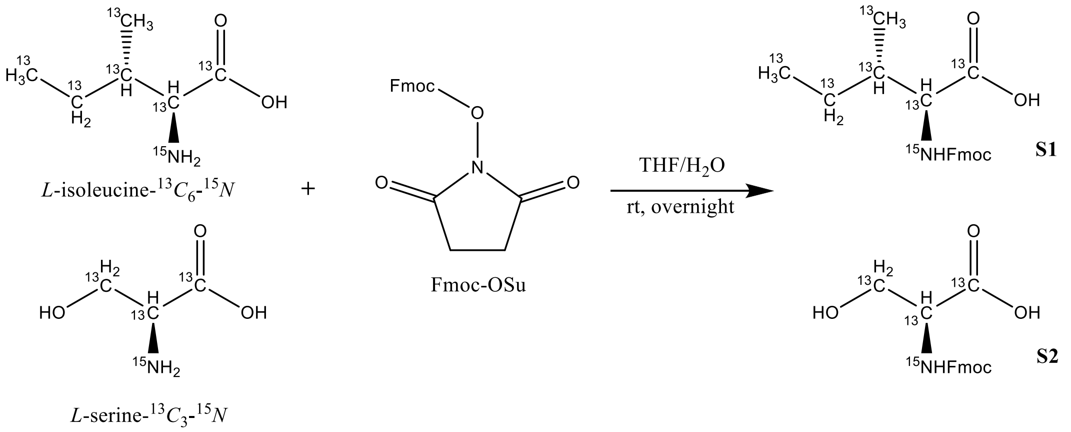

Supplementary Figure 17| Syntheses of Fmoc protected labelled amino acids, S1 and S2

1eq. of labeled L-amino acid (Serine/Isoleucine) was dissolved in 4eq of $NaHCO_3$ in water. Subsequently, a solution of 1.2eq. Fmoc-OSu in THF was added dropwise to the stirring solution of amino acid at room temperature (rt) for 30min. The reaction was further stirred overnight at room temperature. The THF was evaporated under reduced pressure and the aqueous layer was washed with diethyl ether (Et_2O). The aqueous layer was acidified using 2M HCl (pH 2) and then extracted with ethyl acetate (EtOAc) twice. The organic phases were combined and further washed with brine and treated with anhydrous sodium sulphate (Na_2SO_4) and the solvent was evaporated under reduced pressure. The white amorphous solid of Fmoc-amino acids yielded quantitatively and was used without purification.

Fmoc-Ile-OH (compound S1 in Supplementary Figure 17)

^{1}H NMR (500 MHz, Chloroform-*d*) δ 7.76 (d, *J* = 7.6 Hz, 2H), 7.64 – 7.49 (m, 2H), 7.40 (t, *J* = 7.5 Hz, 2H), 7.31 (t, *J* = 7.4 Hz, 2H), 5.30 (dd, *J* = 91.2, 8.8 Hz, 1H), 4.53 (d, *J* = 6.3 Hz, 1H), 4.43 (d, *J* = 7.1 Hz, 2H), 4.23 (t, *J* = 7.1 Hz, 1H), 2.14 – 1.76 (m, 1H), 1.60 (m, 1H), 1.34 (m, 1H), 1.16 – 0.93 (m, 3H), 0.91 – 0.68 (m, 3H).

^{13}C NMR (126 MHz, Chloroform-*d*) *Chemical shifts in red indicate isotopic label ^{13}C.* δ 176.46 (d, *J* = 58.4 Hz), 156.34, 143.78, 141.33, 127.74, 127.09, 125.07, 120.01, 67.10, 58.20 (m), 47.20, 37.71 (q, *J* = 34.7 Hz), 24.88 (t, *J* = 34.8 Hz), 15.50 (d, *J* = 35.3 Hz), 11.60 (d, *J* = 34.7 Hz) (Supplementary Figure 7. 15a). ESI$^+$ mass spectra: calculated for $C_{21}H_{23}NO_4$ ($^{13}C6$, $^{15}N1$,) $[M+H]^+$ calc m/z = 361.16, found m/z = 361.29.

Fmoc-Ser-OH (compound S2 in Supplementary Figure 17)

^{1}H NMR (500 MHz, Chloroform-*d*) δ 7.71 (d, *J* = 7.6 Hz, 2H), 7.54 (t, 7.5 Hz, 2H), 7.34 (t, *J* = 7.6 Hz, 2H), 7.24 (m, 2H), 6.11 (dd, *J* = 92.7, 7.5 Hz, 1H), 4.50 (m, 1H), 4.36 (d, *J* = 7.3 Hz, 1H), 4.30 – 4.10 (m, 2H), 4.04 – 3.61 (m, 2H).

^{13}C NMR (126 MHz, $CDCl_3$) *Chemical shifts in red indicate isotopic label.* δ 173.91-172.68 (m), 156.66, 143.68, 143.52, 141.29, 127.78, 127.11, 125.05, 120.01, 67.42, 63.17-62.15 (m), 56.83-55.11 (m), 46.98 (Supplementary Figure 16b). ESI$^+$ mass spectra: calculated for $C_{18}H_{17}NO_5$ ($^{13}C3$, $^{15}N1$,) $[M+H]^+$ m/z = 332.11, found m/z = 332.29; $[M+Na]^+$ m/z = 354.11, found m/z = 354.23; $[M+Na+H]^+/2$ calc m/z = 177.55, found m/z = 177.26.

References

1 Jekhmane, S. *et al.* Shifts in the selectivity filter dynamics cause modal gating in K+ channels. *Nat Commun* **10**, doi:10.1038/s41467-018-07973-6 (2019).

2 Breukink, E. *et al.* Lipid II is an intrinsic component of the pore induced by nisin in bacterial membranes. *J Biol Chem* **278**, 19898-19903, doi:10.1074/jbc.M301463200 (2003).

3 Weingarth, M., Bodenhausen, G. & Tekely, P. Broadband magnetization transfer using moderate radio-frequency fields for NMR with very high static fields and spinning speeds. *Chem Phys Lett* **488**, 10-16, doi:10.1016/j.cplett.2010.01.072 (2010).

4 Baldus, M. & Meier, B. H. Total correlation spectroscopy in the solid state. The use of scalar couplings to determine the through-bond connectivity. *J Magn Reson Ser A* **121**, 65-69, doi:DOI 10.1006/jmra.1996.0137 (1996).

5 Baldus, M., Iuliucci, R. J. & Meier, B. H. Probing through-bond connectivities and through-space distances in solids by magic-angle-spinning nuclear magnetic resonance. *Journal of the American Chemical Society* **119**, 1121-1124, doi:DOI 10.1021/ja9622259 (1997).

6 Doherty, T. & Hong, M. 2D 1H-31P solid-state NMR studies of the dependence of inter-bilayer water dynamics on lipid headgroup structure and membrane peptides. *J Magn Reson* **196**, 39-47, doi:10.1016/j.jmr.2008.10.001 (2009).

7 Ader, C. *et al.* Structural rearrangements of membrane proteins probed by water-edited solid-state NMR spectroscopy. *J Am Chem Soc* **131**, 170-176, doi:10.1021/ja806306e (2009).

8 Zong, Y. *et al.* Developing Equipotent Teixobactin Analogues against Drug-Resistant Bacteria and Discovering a Hydrophobic Interaction between Lipid II and Teixobactin. *J Med Chem* **61**, 3409-3421, doi:10.1021/acs.jmedchem.7b01241 (2018).

9 Abdel Monaim, S. A. H. *et al.* Lysine Scanning of Arg10-Teixobactin: Deciphering the Role of Hydrophobic and Hydrophilic Residues. *ACS Omega* **1**, 1262-1265, doi:10.1021/acsomega.6b00354 (2016).

10 Chen, K. H., Le, S. P., Han, X., Frias, J. M. & Nowick, J. S. Alanine scan reveals modifiable residues in teixobactin. *Chem Commun (Camb)* **53**, 11357-11359, doi:10.1039/c7cc03415f (2017).

11 Yang, H., Chen, K. H. & Nowick, J. S. Elucidation of the Teixobactin Pharmacophore. *ACS Chem Biol* **11**, 1823-1826, doi:10.1021/acschembio.6b00295 (2016).

12 Parmar, A. *et al.* Defining the molecular structure of teixobactin analogues and understanding their role in antibacterial activities. *Chem Commun* **53**, 2016-2019, doi:10.1039/c6cc09490b (2017).

13 Parmar, A. *et al.* Design and Syntheses of Highly Potent Teixobactin Analogues against Staphylococcus aureus, Methicillin-Resistant Staphylococcus aureus (MRSA), and Vancomycin-Resistant Enterococci (VRE) in Vitro and in Vivo. *J Med Chem* **61**, 2009-2017, doi:10.1021/acs.jmedchem.7b01634 (2018).

14 Ling, L. L. *et al.* A new antibiotic kills pathogens without detectable resistance. *Nature* **517**, 455-459, doi:10.1038/nature14098 (2015).

15 Oster, C. *et al.* Structural studies suggest aggregation as one of the modes of action for teixobactin. *Chem Sci* **9**, 8850-8859, doi:10.1039/c8sc03655a (2018).

16 Yang, H., Wierzbicki, M., Du Bois, D. R. & Nowick, J. S. X-ray Crystallographic Structure of a Teixobactin Derivative Reveals Amyloid-like Assembly. *J Am Chem Soc* **140**, 14028-14032, doi:10.1021/jacs.8b07709 (2018).

17 Shen, Y., Delaglio, F., Cornilescu, G. & Bax, A. TALOS plus : a hybrid method for predicting protein backbone torsion angles from NMR chemical shifts. *J Biomol Nmr* **44**, 213-223, doi:10.1007/s10858-009-9333-z (2009).

18 Jankowski, W. J., Swiezewska, E., Sasak, W. & Chojnacki, T. Occurrence of Polyprenols and Dolichols in Plants. *J Plant Physiol* **143**, 448-452, doi:Doi 10.1016/S0176-1617(11)81806-7 (1994).

19 Parmar, A. *et al.* Teixobactin analogues reveal enduracididine to be non-essential for highly potent antibacterial activity and lipid II binding. *Chem Sci* **8**, 8183-8192, doi:10.1039/c7sc03241b (2017).
